# Immunoglobulin G Antibodies to SARS-CoV-2 Among Healthcare Workers at a Tertiary Care Center in South India

**DOI:** 10.7759/cureus.22520

**Published:** 2022-02-23

**Authors:** Aneesh Basheer, Reba Kanungo, Vivian J Ratnam, Ravichandran Kandasamy

**Affiliations:** 1 General Medicine, Dr. Moopen’s Wayanad Institute of Medical Sciences, Wayanad, IND; 2 Microbiology, Pondicherry Institute of Medical Sciences, Pondicherry, IND; 3 Biostatistics, Pondicherry Institute of Medical Sciences, Pondicherry, IND

**Keywords:** igg antibodies, vaccine, seroprevalence, sars-cov-2, healthcare workers

## Abstract

Introduction

Healthcare workers (HCWs) are at risk of exposure to SARS-CoV-2. Seroprevalence in this group may offer insights into trends to monitor and revise strategies to prevent transmission.

Methods

A cross-sectional study was conducted in two phases among healthcare workers at a tertiary care center to detect IgG antibodies to SARS-CoV-2. Seropositivity was calculated during both phases, and possible associations were determined using regression analysis.

Results

A total of 382 and 168 HCWs took part in the two phases, respectively. IgG antibodies were detected in 13 of 382 (3.4%; 95% confidence interval (CI): 2%-5.7%) and 71 of 168 (42.3%) participants in the first and second phases, respectively. Receiving at least one dose of vaccine (p < 0.001) and age (p = 0.028) were factors associated with the presence of antibodies, while gender, job type, exposure to COVID-19 cases, and comorbidities were not associated with seropositivity.

Conclusion

Serosurveys among HCWs may help identify transmission patterns and redesign infection control practices in the healthcare setting.

## Introduction

Our understanding of the immune response to SARS-CoV-2 has undergone significant progress over the course of the pandemic. This has helped in establishing antibody detection assays to detect past exposures to the virus, screen for epidemiological surveys, and detect response to vaccines [1]. Antibody response to SARS-CoV-2 has mostly been studied in vitro, showing a peak IgM response during the second week after infection and IgG response much later by around 20 days [2]. The clinical significance of these antibodies is still unclear. The presence of IgG antibodies indicates that the individual has been exposed to the virus. Few studies have shown that higher IgG levels were related to recovery and that high titers of IgM were related to rapid decline or death [3]. Nevertheless, it is logical to believe that IgG antibodies indicate exposure to the virus, thereby providing information about the disease transmission in a given population. However, its direct relation to protection remains a challenge in the non-vaccinated population, unless neutralizing antibodies are specifically examined. Healthcare workers (HCWs) are at the forefront of the battle against COVID-19. Studies on HCWs have evaluated the antibodies in RT-PCR-positive confirmed cases, while we now know that asymptomatic infection is quite common. They are at very high risk of infection since they are frequently exposed to symptomatic and asymptomatic individuals and often have to undertake lifesaving interventions, exposing them to a high risk of transmission. It is believed that if HCWs demonstrate antibodies in their blood, it would indicate that they have been exposed to the virus and/or vaccine. This will help in formulating policies on further streamlining their preventive and protective protocols. With the potential of HCWs to exposure and subsequent infection, it becomes essential to determine their status of exposure to the virus. This study was planned to detect IgG antibodies to SARS-CoV-2 among HCWs who have not been diagnosed with COVID-19 disease.

## Materials and methods

This was a cross-sectional study conducted at Pondicherry Institute of Medical Sciences, a tertiary care teaching hospital designated as a testing and treatment center for COVID-19. This study was reviewed and approved by the Institute Ethics Committee. The study included hospital staff comprising of senior and junior doctors, nursing staff, technical staff dealing with samples from COVID-19 patients, and housekeeping staff involved in biomedical waste disposal in the hospital. Individuals who had active symptoms of COVID-19 were excluded. Following the identification of all eligible volunteers, we used stratified random sampling to ensure the representation of HCWs from all categories of job designations and exposures. After obtaining written informed consent, the participants were interviewed to obtain demographic details including age, type of work, degree of exposure to patients as part of their job, geographic area of residence (hot spot/containment zone currently or in the past one month), and any potential contact with confirmed/suspect patients in the hospital, household, or immediate work area. In participants who had a contact history, we also collected details of personal protective equipment (PPE) use during such encounters. Assuming that 10% of the HCWs would have IgG antibodies against SARS-CoV-2 in a high prevalence setting such as ours, with 99% confidence limits and 5% absolute precision, 250 participants were required for the study. However, we included all 382 HCWs who volunteered for the study in the first phase that happened between July 2020 and September 2020. Subsequently, from February 2021 to April 2021, a second sampling was done on 168 participants to determine any change in seroprevalence among HCWs during the intervening period. None of these participants had taken part in the first phase.

Using aseptic techniques, 2 mL of venous blood was collected in vacutainers. The samples were processed in the Microbiology department and tested for IgG by Elecsys Anti-SARS-CoV-2 Kit procured from Roche Diagnostics (Rotkreuz, Switzerland). The test was performed in a Cobas e 411 analyzer. Patient sera were incubated with a mix of biotinylated and ruthenylated nucleocapsid (N) antigens. The principle is of double-antigen sandwich immune complexes, which are formed in the presence of corresponding antibodies. Streptavidin-coated microparticles were added, and the double-antigen sandwich immune complexes bound to the solid phase via the interaction of biotin and streptavidin. The reagent mixture was then transferred to the measuring cell, where the microparticles were magnetically captured onto the surface of the electrode. Unbound substances were subsequently removed. Electrochemiluminescence was then induced by applying a voltage and measured with a photomultiplier. The signal yield increases with the antibody titer. The results were displayed as positive or negative. Samples with a cutoff index of <1.0 were considered negative, and samples with a cutoff index of ≥1.0 were considered positive (as per the kit insert).

The seroprevalence of IgG antibodies was calculated and expressed as percentages with confidence intervals. The Shapiro-Wilk test was done to check for normality. The Chi-square test/Fisher’s exact test was used to compare proportions and the Mann-Whitney test for the comparison of continuous variables. Regression analysis was employed to estimate the odds ratios of demographic and other variables potentially associated with IgG antibody positivity. p values of less than 0.05 were used to define statistical significance. All analyses were done in SPSS version 20.0 (IBM Corporation, Armonk, NY, USA).

## Results

In the first phase, 382 HCWs were included in the study. Of these, 180 (47.1%) were doctors, 69 (18.1%) were nurses, 19 (5%) were laboratory personnel, and 114 (29.8%) were ancillary staff. The mean age of the HCWs was 33.4 (SD: 10.1) years. Of the 382 participants, 130 (34%) HCWs were males, 248 (64.9%) worked in areas with direct patient interaction, 91 (23.8%) had a history of contact with patient specimens with high risks such as aerosols or sputum, and 380 (99.5%) had contact with suspected COVID-19 patients in the last three months; of these, 274 (71.7%) were not using personal protective equipment at the time of contact. Out of 382 HCWs, 166 (43.5%) had contact with a confirmed case of COVID-19 in the last three months in either hospital or community setting; 104 of these 166 (62.7%) were using exposure-specified PPE at the time of contact. HCWs’ area of usual work, their baseline sociodemographic details, and comorbidities are summarized in Table 1.

**Table 1:** Distribution of the sociodemographic and clinical characteristics of the study participants in the first and second phases * Total will not add up to 168 for most of the variables as complete information was not available for three participants ** Median and interquartile range @ Total will be more as a person will be working in more than one area

Characteristics	First phase (n = 382)	Second phase (n = 168)*
	Number (%)	Number (%)
Age**	30 (26–39)	40 (31–48)
Male	130 (34)	65 (38.7)
Residence at a hot spot	129 (33.8)	2 (1.2)
Designation		
Doctors	180 (47.1)	128 (76.2)
Nurses	69 (18.1)	12 (7.1)
Laboratory personnel	19 (5)	4 (2.4)
Others	114 (29.8)	21 (12.5)
Area of usual work^@^		
OPD	186 (48.7)	91 (54.2)
Ward	143 (37.4)	57 (33.9)
OT	64 (16.8)	49 (29.2)
Emergency	62 (16.2)	35 (20.8)
Office	28 (7.3)	30 (17.9)
Residence at a containment zone	91 (23.8)	1 (0.6)
Nature of work^@^		
Direct patient interaction	248 (64.9)	96 (57.1)
Indirect patient interaction	33 (8.6)	18 (10.7)
Contact with patient specimen	91 (23.8)	55 (32.7)
H/O contact with suspected COVID-19 patient	380 (99.5)	73 (43.5)
H/O contact with confirmed COVID-19 patient	166 (43.5)	66 (39.3)
COVID Antibody Positive	13 (3.4)	71 (42.3)

Diabetes and hypertension were the two major comorbidities in 2.1% and 1.9% of HCWs, respectively, while five healthcare workers had chronic lung disease (asthma), and only one person had chronic kidney disease. Fever (3.4% and 2.4%), cough (3.7% and 4.2%), and runny nose (4.7% and 4.2%) were present almost equally among the personnel in the first and second phases, respectively. Overall, 13 of the 382 (3.4%; 95% CI: 2%-5.7%) HCWs had IgG antibodies to SARS-CoV-2 in the first phase. None of the doctors tested had antibodies, while nine of the 69 (13%) nurses and two of the 19 (10.5%) laboratory staff had detectable IgG antibodies against SARS-CoV-2. Younger age (p = 0.029), nursing job (p < 0.001), and residence in a containment zone (p = 0.017) were associated with the presence of IgG antibodies (Table 2).

**Table 2:** Factors associated with the presence of IgG antibodies in the first and second phases * For most variables, information was not available for three participants ** Median and interquartile range @ Continuous variables, Mann–Whitney test; categorical variable: Chi-square/Fisher’s exact test NA: not applicable

Variables	First phase (n = 382)			Second phase (n = 168)*		
	Positive (n = 13)	Negative (n = 369)	p value^@^	Positive (n = 71)	Negative (n = 97)	p value^@^
Age**	26 (24–30)	31 (26–39)	0.029	43 (33–58)	37 (30–46)	0.028
Gender						
Male	5	125	0.770	29	36	0.557
Female	8	244		40	60	
Designation						
Doctors	0	180	< 0.001	56	72	0.082
Nurses	9	60		7	5	
Laboratory personnel	2	17		2	2	
Others	2	112		4	17	
Residence at a hot spot						
Yes	3	126	0.556	1	1	1.000
No	10	243		68	95	
Residence at a containment zone						
Yes	7	84	0.017	1	0	0.418
No	6	285		68	96	
H/O contact with confirmed COVID-19 patient						
Yes	8	158	0.181	25	41	0.654
No	5	211		43	53	
Vaccination						
Yes	NA	NA	NA	28	94	< 0.001
No	NA	NA	NA	43	3	

**Figure 1:**
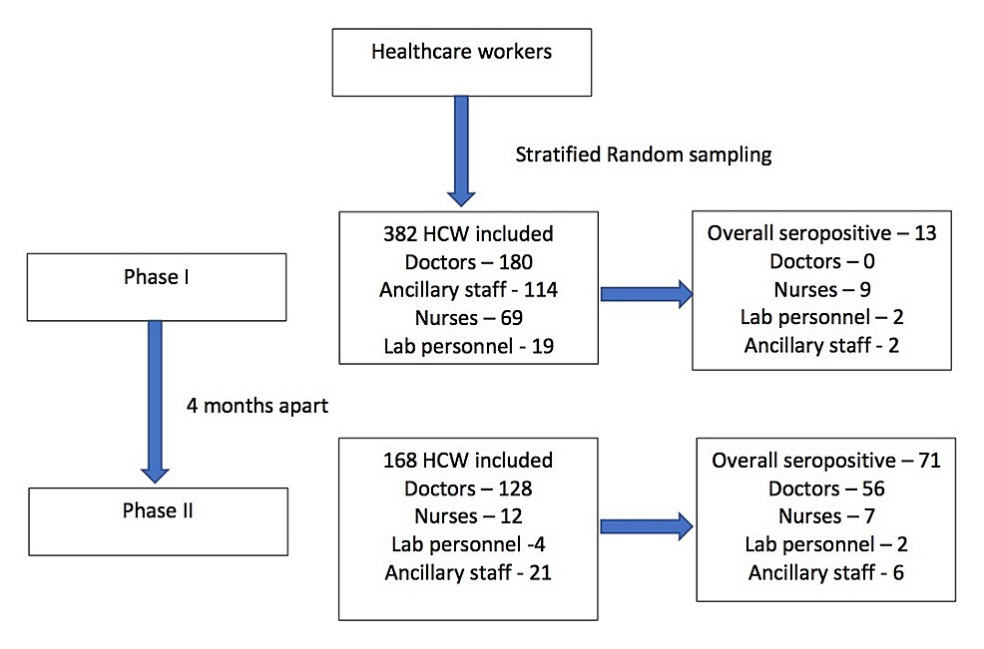
Participant enrollment and seropositivity status by categories of HCWs in the first and second phases of the study

In the second phase of the study, the samples were obtained after a gap of four months. By then, several of the participants of the first phase had been infected. Of the 168 HCWs who volunteered, 71 (42.3%) had IgG antibodies to SAR-CoV-2. This included 56 doctors, seven nurses, two laboratory personnel, and six ancillary staff. Of the 168 participants in the second phase, 122 (72.6%) were unvaccinated, while the remaining 46 (27.4%) had received at least one dose of vaccination. A total of 96 (57.1%) had worked in areas with direct patient interaction, while 55 (32.7%) had contact with patient specimens. There was a history of contact with suspected COVID-19 patients in 73 of 168 (43.5%) HCWs, and 66 (39.3%) HCWs had contact with confirmed cases of COVID-19 in the last three months. None of the variables gender, designation, contact with a suspected case, and contact with a confirmed case of COVID-19 were associated with the presence of antibodies. Receiving one or both doses of vaccine (p < 0.001) and older age (p = 0.028) were the factors associated with the presence of antibodies (Table 3). A flow diagram of the sampling and major results of phases 1 and 2 are provided in Figure 1.

**Table 3:** Univariate regression analysis of factors associated with the presence of IgG antibodies * Not applicable for the first phase

Variables	First phase			Second phase		
	Odds ratio	95% CI	p value	Odds ratio	95% CI	p value
Age	0.895	0.809–0.991	0.033	1.033	1.008–1.059	0.010
Vaccination*						
Yes	-	-	-	1.00	-	-
No	-	-	-	48.119	13.867–166.971	< 0.001

## Discussion

This cross-sectional study on healthcare workers demonstrated that there has been a significant increase in the seroprevalence of IgG antibodies to SARS-CoV-2 following the first wave of COVID-19. However, neither exposure to confirmed/suspected cases nor comorbidities had any association with the presence of antibodies. Vaccination was the only factor associated with the development of antibodies.

This study was initiated when the union territory of Pondicherry was reporting very few cases. Hence, it is obvious that the prevalence of antibodies was low among HCWs as well. A study from Belgium around that time identified that 6.4% of the hospital staff had IgG antibodies to SARS-CoV-2 (95% CI: 5.5%-7.3%) [4], while another from Japan showed a low number of SARS-CoV-2-seropositive HCWs in a low-prevalence setting [5], reflecting the variability in seroprevalence paralleling the diversity in caseload among the general population. However, in our study, subsequently following the end of the first wave, the seroprevalence increased substantially (42.3%). This increase was remarkable and higher than the general population seroprevalence of 26% during that period in India [6]. While the risk of exposure is higher among HCWs, such a wide gap in seropositivity between HCWs and the general population is difficult to explain given the well-streamlined infection control practices in place by this time of the pandemic. In the present study, adherence to strict COVID-19-specific infection control practices, such as wearing of PPE and mandatory testing of patients, had not been implemented in the early days of the pandemic, leading to exposure of HCWs to possible COVID-19-positive cases. The other possible explanation is that exposure to the virus had become almost universal by then, breaking all barriers including the hospital environment.

Serological testing is believed to complement nasopharyngeal swab testing to detect the true burden of SARS-CoV-2 infection. In specific high-risk groups such as HCWs, it may offer insights into the transmission patterns and risks of exposure. The findings of this study differ from the Indian Council of Medical Research (ICMR) national serosurveillance data that found serological evidence of infection in 25.7% of HCWs in December 2020 [6]. Our data collected at around the same time shows 42.3% seroprevalence among HCWs. In general, most studies from different parts of the world on seroprevalence among HCWs during that time reported lower prevalence ranging from 0.34% in Japan to 13.7% in New York [7,8]. A recent systematic review reported seroprevalence of 8.7% among HCWs - much lower than our study and the national serosurvey findings [9]. Despite the high caseload in countries such as the United States and Europe, HCWs seemed to have lesser serological evidence of infection [9]. While this is probably a reflection of better implementation and adherence to personal protection protocols, the disparity may not be completely explained by this since PPE availability and infection control practices had become streamlined by then in most parts of the world, including India.

In general, more doctors in the present study had antibodies compared to other HCWs; this could be due to sampling issues in the second phase. However, the study did not find any difference in seropositivity among different categories of HCWs. This is similar to the findings of the third national serosurvey conducted by the ICMR [6]. These findings reiterate the widespread exposure to SARS-CoV-2 in the hospital environment, irrespective of the type of exposure, and probably reflect how the virus has become a universal presence.

Most HCWs had a history of contact with suspected or confirmed cases of COVID-19, although this was not associated with positivity. The ICMR survey showed higher seroprevalence among HCWs who reported contact with COVID-19 cases.

In our study, the seropositivity did not differ by gender. The national serosurvey results also showed similar findings [6,10]. However, age was associated with seropositivity. Interestingly, seropositivity was associated with younger age in the first phase and older age in the second. While this could partly be due to sampling issues in the second phase of our study, this association has been inconsistent across other studies as well [11-13]. Vaccination was associated with seropositivity and contributed partly to the increase in the number of HCWs with antibodies in the second phase of the study. Out of 71 HCWs who participated in both phases, 28 (39.4%) were positive prior to their vaccination and 43 (60.6%) post-vaccination. This emphasizes the role of vaccination among HCWs being a high-risk group, such as other high-risk groups in the general population. However, we are not sure to what extent vaccination contributed to the increase in seropositivity within this short intervening span of four months.

Our study also underlines the importance of repeated cross-sectional surveys in the same area and specific subgroups. Such repeated surveys help monitor trends over a period of time and enable designing or revising strategies to contain transmission of infection [14,15].

Seroprevalence studies among HCWs are all the more important as it provides information on the risk of transmission of infection within a hospital, as well as evaluation of infection control practices and adherence to protocols [16].

This study had several limitations. First, the second phase was conducted during a time when vaccination was being rolled out, and hence, seropositivity may have been contributed partly by this. Therefore, the seropositivity following exposure to virus might be actually lower than reported. Second, the sample size for the second phase was lower since many HCWs had already been infected by then and did not consent to take part. Third, we were also unable to detect specific neutralizing antibodies against SARS-CoV-2. Finally, we could not determine the reasons for very high seropositivity in the second phase despite following preventive protocols and stringent hospital infection control measures.

## Conclusions

Serosurveillance among healthcare workers is important for monitoring trends of COVID-19 infection and exposure rates. It is crucial to develop, modify, and make policy decisions on infection control practices in hospitals. There was a significant increase in seropositivity among healthcare workers in the second phase compared to the first phase. Vaccination is strongly associated with seropositivity and underlines the need for prioritizing vaccination among healthcare workers in a pandemic such as this. More studies are needed to determine the utility of repeated cross-sectional serosurveys among healthcare workers in infection control and pandemic containment.
